# Supplementary material for: Fast and scalable likelihood maximization for Exponential Random Graph Models with local constraints
Source: Sci Rep. 2021 Jul 27;11:15227. doi: 10.1038/s41598-021-93830-4 (PMC8316481; doi:10.1038/s41598-021-93830-4)
Supplement: Supplementary file 1 — Supplementary Information. [file 41598_2021_93830_MOESM1_ESM.pdf]

Fast and scalable likelihood maximization for Exponential Random Graph Models with local constraints

Nicolò Vallarano,[1] Matteo Bruno,[1] Emiliano Marchese,[1] Giuseppe Trapani,[1] Fabio Saracco,[1] Giulio Cimini,[2] Mario Zanon,[1] and Tiziano Squartini[1,3,*]

[1] *IMT School for Advanced Studies Lucca, P.zza San Francesco 19, 55100 Lucca (Italy)*
[2] *Physics Department and INFN, 'Tor Vergata' University of Rome, 00133 Rome (Italy)*
[3] *Institute for Advanced Study (IAS), University of Amsterdam, Oude Turfmarkt 145, 1012 GC Amsterdam (The Netherlands)*

(Dated: July 9, 2021)

Abstract. Supplementary Information file for the article "Fast and scalable likelihood maximization for Exponential Random Graph Models".

PACS numbers: 02.10.Ox, 89.75.Hc, 02.70.Rr, 05.10.-a

A: COMPUTING THE HESSIAN MATRIX

As we showed in the main text, the Hessian matrix of our likelihood function is 'minus' the covariance matrix of the constraints, i.e.

$$H_{ij}=\frac{\partial^2\mathscr{L}(\vec{\theta})}{\partial\theta_i\partial\theta_j}=-\text{Cov}[C_i,C_j],\quad i,j=1\dots M; \tag{1}$$

interestingly, a variety of alternative methods exists to explicitly calculate the generic entry H_{ij}, i.e. 1) taking the second derivatives of the likelihood function characterizing the method under analysis, 2) taking the first derivatives of the expectation values of the constraints characterizing the method under analysis, 3) calculating the moments of the pair-specific probability distributions characterizing each method.

UBCM: binary undirected graphs with given degree sequence

The Hessian matrix for the UBCM is an $N\times N$ symmetric table with entries reading

$$H_{\text{UBCM}}=\begin{cases}\text{Var}[k_i]=\sum_{\substack{j=1\\(j\neq i)}}^N p_{ij}(1-p_{ij}), & \forall\, i\\ \text{Cov}[k_i,k_j]=p_{ij}(1-p_{ij}), & \forall\, i\neq j\end{cases} \tag{2}$$

where $p_{ij}\equiv p_{ij}^{\text{UBCM}}$. Notice that $\text{Var}[k_i]=\sum_{\substack{j=1\\(j\neq i)}}^N \text{Cov}[k_i,k_j]$, $\forall\, i$.

DBCM: binary directed graphs with given in-degree and out-degree sequences

The Hessian matrix for the DBCM is a $2N\times 2N$ symmetric table that can be further subdivided into four $N\times N$ blocks whose entries read

$$H_{\text{DBCM}}=\begin{cases}\text{Var}[k_i^{out}]=\sum_{\substack{j=1\\(j\neq i)}}^N p_{ij}(1-p_{ij}), & \forall\, i\\ \text{Var}[k_i^{in}]=\sum_{\substack{j=1\\(j\neq i)}}^N p_{ji}(1-p_{ji}), & \forall\, i\\ \text{Cov}[k_i^{out},k_j^{in}]=p_{ij}(1-p_{ij}), & \forall\, i\neq j\\ \text{Cov}[k_j^{out},k_i^{in}]=p_{ji}(1-p_{ji}), & \forall\, i\neq j\end{cases} \tag{3}$$

while $\text{Cov}[k_i^{out},k_i^{in}]=\text{Cov}[k_i^{out},k_j^{out}]=\text{Cov}[k_i^{in},k_j^{in}]=0$ and $p_{ij}\equiv p_{ij}^{\text{DBCM}}$.

Notice that the Hessian matrix of the BiCM mimicks the DBCM one, the only difference being that the probability coefficients are now indexed by i and α: for example, in the BiCM case, one has that $\text{Cov}[k_i,d_\alpha]=p_{i\alpha}(1-p_{i\alpha})$, $\forall\, i,\alpha$.

UECM: weighted undirected graphs with given strengths and degrees

The Hessian matrix for the UECM is a $2N\times 2N$ symmetric table that can be further subdivided into four blocks (each of which with dimensions $N\times N$). In order to save space, the expressions indexed by the single subscript i will be assumed as being valid $\forall\, i$, while the ones indexed by a double subscript i,j will be assumed as being valid $\forall\, i\neq j$. The entries of the diagonal blocks read

$$H_{\text{UECM}}=\begin{cases}\frac{\partial^2\mathscr{L}_{\text{UECM}}}{\partial\alpha_i^2}=\text{Var}[k_i]=\sum_{\substack{j=1\\(j\neq i)}}^N p_{ij}(1-p_{ij})\\ \frac{\partial^2\mathscr{L}_{\text{UECM}}}{\partial\alpha_i\alpha_j}=\text{Cov}[k_i,k_j]=p_{ij}(1-p_{ij})\end{cases} \tag{4}$$

and

$$H_{\rm UECM}=\begin{cases}\frac{\partial^2\mathscr{L}_{\rm UECM}}{\partial\beta_i^2}=\text{Var}[s_i]=\sum_{\substack{j=1\\(j\neq i)}}^N\frac{p_{ij}(1-p_{ij}+e^{-\beta_i-\beta_j})}{(1-e^{-\beta_i-\beta_j})^2}\\ \frac{\partial^2\mathscr{L}_{\rm UECM}}{\partial\beta_i\beta_j}=\text{Cov}[s_i,s_j]=\frac{p_{ij}(1-p_{ij}+e^{-\beta_i-\beta_j})}{(1-e^{-\beta_i-\beta_j})^2}\end{cases}\tag{5}$$

where $p_{ij}\equiv p_{ij}^{\rm UECM}$. On the other hand, the entries of the off-diagonal blocks read

$$H_{\rm UECM}=\begin{cases}\frac{\partial^2\mathscr{L}_{\rm UECM}}{\partial\alpha_i\partial\beta_i}=\text{Cov}[k_i,s_i]=\sum_{\substack{j=1\\(j\neq i)}}^N\frac{p_{ij}(1-p_{ij})}{1-e^{-\beta_i-\beta_j}}\\ \frac{\partial^2\mathscr{L}_{\rm UECM}}{\partial\alpha_i\partial\beta_j}=\text{Cov}[k_i,s_j]=\frac{p_{ij}(1-p_{ij})}{1-e^{-\beta_i-\beta_j}}\end{cases}\tag{6}$$

with $p_{ij}\equiv p_{ij}^{\rm UECM}$.

DECM: weighted directed graphs with given strengths and degrees

The Hessian matrix for the DECM is a $4N\times 4N$ symmetric table that can be further subdivided into four blocks (each of which with dimensions $N\times N$). As for the UECM, in order to save space, the expressions indexed by the single subscript i will be assumed as being valid $\forall\, i$, while the ones indexed by a double subscript i,j will be assumed as being valid $\forall\, i\neq j$. The entries of the diagonal blocks read

$$H_{\rm DECM}=\begin{cases}\frac{\partial^2\mathscr{L}_{\rm DECM}}{\partial\alpha_i^2}=\text{Var}[k_i^{out}]=\sum_{\substack{j=1\\(j\neq i)}}^N p_{ij}(1-p_{ij})\\ \frac{\partial^2\mathscr{L}_{\rm DECM}}{\partial\alpha_i\alpha_j}=\text{Cov}[k_i^{out},k_j^{out}]=0\end{cases}\tag{7}$$

and

$$H_{\rm DECM}=\begin{cases}\frac{\partial^2\mathscr{L}_{\rm DECM}}{\partial\beta_i^2}=\text{Var}[k_i^{in}]=\sum_{\substack{j=1\\(j\neq i)}}^N p_{ji}(1-p_{ji})\\ \frac{\partial^2\mathscr{L}_{\rm DECM}}{\partial\beta_i\beta_j}=\text{Cov}[k_i^{in},k_j^{in}]=0\end{cases}\tag{8}$$

and

$$H_{\rm DECM}=\begin{cases}\frac{\partial^2\mathscr{L}_{\rm DECM}}{\partial\gamma_i^2}=\text{Var}[s_i^{out}]=\sum_{\substack{j=1\\(j\neq i)}}^N\frac{p_{ij}(1-p_{ij}+e^{-\gamma_i-\delta_j})}{(1-e^{-\gamma_i-\delta_j})^2}\\ \frac{\partial^2\mathscr{L}_{\rm DECM}}{\partial\gamma_i\gamma_j}=\text{Cov}[s_i^{out},s_j^{out}]=0\end{cases}\tag{9}$$

and

$$H_{\rm DECM}=\begin{cases}\frac{\partial^2\mathscr{L}_{\rm DECM}}{\partial\delta_i^2}=\text{Var}[s_i^{in}]=\sum_{\substack{j=1\\(j\neq i)}}^N\frac{p_{ji}(1-p_{ji}+e^{-\gamma_j-\delta_i})}{(1-e^{-\gamma_j-\delta_i})^2}\\ \frac{\partial^2\mathscr{L}_{\rm DECM}}{\partial\delta_i\delta_j}=\text{Cov}[s_i^{in},s_j^{in}]=0\end{cases}\tag{10}$$

where $p_{ij}\equiv p_{ij}^{\rm DECM}$. On the other hand, the entries of the off-diagonal blocks read

$$H_{\rm DECM}=\begin{cases}\frac{\partial^2\mathscr{L}_{\rm DECM}}{\partial\alpha_i\partial\beta_i}=\text{Cov}[k_i^{out},k_i^{in}]=0\\ \frac{\partial^2\mathscr{L}_{\rm DECM}}{\partial\alpha_i\partial\beta_j}=\text{Cov}[k_i^{out},k_j^{in}]=p_{ij}(1-p_{ij})\end{cases}\tag{11}$$

and

$$H_{\rm DECM}=\begin{cases}\frac{\partial^2\mathscr{L}_{\rm DECM}}{\partial\alpha_i\partial\gamma_i}=\text{Cov}[k_i^{out},s_i^{out}]=\sum_{\substack{j=1\\(j\neq i)}}^N\frac{p_{ij}(1-p_{ij})}{1-e^{-\gamma_i-\delta_j}}\\ \frac{\partial^2\mathscr{L}_{\rm DECM}}{\partial\alpha_i\partial\gamma_j}=\text{Cov}[k_i^{out},s_j^{out}]=0\end{cases}\tag{12}$$

and

$$H_{\rm DECM}=\begin{cases}\frac{\partial^2\mathscr{L}_{\rm DECM}}{\partial\alpha_i\partial\delta_i}=\text{Cov}[k_i^{out},s_i^{in}]=0\\ \frac{\partial^2\mathscr{L}_{\rm DECM}}{\partial\alpha_i\partial\delta_j}=\text{Cov}[k_i^{out},s_j^{in}]=\frac{p_{ij}(1-p_{ij})}{1-e^{-\gamma_i-\delta_j}}\end{cases}\tag{13}$$

and

$$H_{\rm DECM}=\begin{cases}\frac{\partial^2\mathscr{L}_{\rm DECM}}{\partial\beta_i\partial\gamma_i}=\text{Cov}[k_i^{in},s_i^{out}]=0\\ \frac{\partial^2\mathscr{L}_{\rm DECM}}{\partial\beta_i\partial\gamma_j}=\text{Cov}[k_i^{in},s_j^{out}]=\frac{p_{ji}(1-p_{ji})}{1-e^{-\gamma_j-\delta_i}}\end{cases}\tag{14}$$

and

$$H_{\rm DECM}=\begin{cases}\frac{\partial^2\mathscr{L}_{\rm DECM}}{\partial\beta_i\partial\delta_i}=\text{Cov}[k_i^{in},s_i^{in}]=\sum_{\substack{j=1\\(j\neq i)}}^N\frac{p_{ji}(1-p_{ji})}{1-e^{-\gamma_j-\delta_i}}\\ \frac{\partial^2\mathscr{L}_{\rm DECM}}{\partial\beta_i\partial\delta_j}=\text{Cov}[k_i^{in},s_j^{in}]=0\end{cases}\tag{15}$$

and

$$H_{\rm DECM}=\begin{cases}\frac{\partial^2\mathscr{L}_{\rm DECM}}{\partial\gamma_i\partial\delta_i}=\text{Cov}[s_i^{out},s_i^{in}]=0\\ \frac{\partial^2\mathscr{L}_{\rm DECM}}{\partial\gamma_i\partial\delta_j}=\text{Cov}[s_i^{out},s_j^{in}]=\frac{p_{ij}(1-p_{ij}+e^{-\gamma_i-\delta_j})}{(1-e^{-\gamma_i-\delta_j})^2}\end{cases}\tag{16}$$

with $p_{ij}\equiv p_{ij}^{\rm DECM}$.

Two-step models for undirected and directed networks

The Hessian matrix for the undirected two-step model considered here is an $N\times N$ symmetric table reading

$$H_{\rm CReM}^{\rm und}=\begin{cases}\text{Var}[s_i]=\sum_{\substack{j=1\\(j\neq i)}}^N\frac{f_{ij}}{(\theta_i+\theta_j)^2},\quad\forall\, i\\ \text{Cov}[s_i,s_j]=\frac{f_{ij}}{(\theta_i+\theta_j)^2},\quad\forall\, i\neq j\end{cases}\tag{17}$$

where f_{ij} is given. In the directed case, instead, the Hessian matrix for the two-step model considered here is a $2N\times 2N$ symmetric table that can be further subdivided into four $N\times N$ blocks whose entries read

$$H_{\rm CReM}^{\rm dir}\begin{cases}{\rm Var}[s_i^{out}]=\sum_{\substack{j=1\\(j\neq i)}}^N\frac{f_{ij}}{(\alpha_i+\beta_j)^2}, & \forall\, i\\ {\rm Var}[s_i^{in}]=\sum_{\substack{j=1\\(j\neq i)}}^N\frac{f_{ji}}{(\alpha_j+\beta_i)^2}, & \forall\, i\\ {\rm Cov}[s_i^{out},s_j^{in}]=\frac{f_{ij}}{(\alpha_i+\beta_j)^2}, & \forall\, i\neq j\end{cases}\qquad(18)$$

while ${\rm Cov}[s_i^{out},s_i^{in}]={\rm Cov}[s_i^{out},s_j^{out}]={\rm Cov}[s_i^{in},s_j^{in}]=0$ and f_{ij} is given.

B: A NOTE ON THE CHANGE OF VARIABLES

In all methods we will considered in the present work, the variable θ_i appears in the optimality conditions only through negative exponential functions: it is therefore tempting to perform the change of variable $x_i\equiv e^{-\theta_i}$. Although this is often performed in the literature, one cannot guarantee that the new optimization problem remains convex: in fact, simple examples can be provided for which convexity is lost. This has several consequences, e.g. 1) convergence to the global maximum is no longer guaranteed (since the existence of a global maximum is no longer guaranteed as well), 2) extra-care is needed to guarantee that the Hessian matrix $\mathbf{H}$ employed in our algorithms is negative definite. While problem 2) introduces additional complexity only for Newton's method, problem 1) is more serious from a theoretical point of view.

Let us now address problem 1) in more detail. First, it is possible to prove that any stationary point for $\mathscr{L}(\vec{x})$ satisfies the optimality conditions for $\mathscr{L}(\vec{\theta})$ as well. In fact, the application of the 'chain rule' leads to recover the set of relationships

$$\frac{\partial\mathscr{L}(\vec{\theta})}{\partial\theta_i}=\frac{\partial x_i}{\partial\theta_i}\frac{\partial\mathscr{L}(\vec{x})}{\partial x_i}=-x_i\frac{\partial\mathscr{L}(\vec{x})}{\partial x_i},\quad i=1\dots M;\qquad(19)$$

notice that requiring $\nabla_{\theta_i}\mathscr{L}(\vec{\theta})=0$ leads to require that either $\nabla_{x_i}\mathscr{L}(\vec{x})=0$ or $x_i=0$. As the second eventuality precisely identifies *isolated* nodes (i.e. the nodes for which the constraint $C_i(\mathbf{G}^*)$, controlled by the multiplier θ_i, is 0), one can get rid of it by explicitly removing the corresponding addenda from the likelihood function.

For what concerns convexity, let us explicitly calculate the Hessian matrix for the set of variables $\{x_i\}_{i=1}^M$. In formulas,

$$\frac{\partial^2\mathscr{L}(\vec{x})}{\partial x_i^2}=e^{2\theta_i}\left(\frac{\partial^2\mathscr{L}(\vec{\theta})}{\partial\theta_i^2}+\frac{\partial\mathscr{L}(\vec{\theta})}{\partial\theta_i}\right),\quad i=1\dots M,$$
$$\frac{\partial^2\mathscr{L}(\vec{x})}{\partial x_i\partial x_j}=e^{\theta_i+\theta_j}\left(\frac{\partial^2\mathscr{L}(\vec{\theta})}{\partial\theta_i\partial\theta_j}\right),\quad\forall\, i\neq j\qquad(20)$$

according to the 'chain rule' for second-order derivatives. More compactly,

$$\mathbf{H}_{\mathscr{L}(\vec{x})}=e^{\Theta}\circ\left(-{\rm Cov}[C_i,C_j]+\mathbf{I}\cdot\nabla_{\vec{\theta}}\mathscr{L}(\vec{\theta})\right)\qquad(21)$$

where $\mathbf{I}$ is the identity matrix, the generic entry of the matrix e^{Θ} reads $\left[e^{\Theta}\right]_{ij}\equiv e^{\theta_i+\theta_j}$, $\forall\, i,j$ and the symbol '$\circ$' indicates the Hadamard (i.e. element-wise) product of matrices. In general, the expression above defines an *indefinite* matrix, i.e. a neither positive nor negative (semi)definite one.

C: FIXED POINT METHOD IN THE MULTIVARIATE CASE

We can rewrite equation (21) at page 4 of the main article as:

$$\theta_i^{(n)}=G_i(\vec{\theta}^{(n-1)}),\quad i=1\dots N;\qquad(22)$$

for the sake of illustration, let us discuss it for the UBCM case. In this particular case, the set of equations above can be rewritten as

$$\theta_i^{(n)}=-\ln\left[\frac{k_i(\mathbf{A}^*)}{\sum_{\substack{j=1\\(j\neq i)}}^N\left(\frac{e^{-\theta_j^{(n-1)}}}{1+e^{-\theta_i^{(n-1)}-\theta_j^{(n-1)}}}\right)}\right],\quad i=1\dots N.\qquad(23)$$

Since all components of the map $\mathbf{G}$ are continuous on $\mathbb{R}^N$, the map itself is continuous on $\mathbb{R}^N$. Hence, a fixed point exists. Let us now consider its Jacobian matrix and check the magnitude of its elements. In the UBCM case, one finds that

$$\frac{\partial G_i}{\partial\theta_i}=\frac{\sum_{\substack{j=1\\(j\neq i)}}^N\frac{e^{-\theta_i-2\theta_j}}{\left(1+e^{-\theta_i-\theta_j}\right)^2}}{\sum_{\substack{j=1\\(j\neq i)}}^N\left(\frac{e^{-\theta_j}}{1+e^{-\theta_i-\theta_j}}\right)}=\frac{\sum_{\substack{j=1\\(j\neq i)}}^N\left(\frac{e^{-\theta_i-\theta_j}}{1+e^{-\theta_i-\theta_j}}\right)^2}{\sum_{\substack{j=1\\(j\neq i)}}^N\left(\frac{e^{-\theta_i-\theta_j}}{1+e^{-\theta_i-\theta_j}}\right)}$$
$$=1-\frac{{\rm Var}[k_i]}{\langle k_i\rangle}=1-\frac{\sum_{j\neq i}{\rm Cov}[k_i,k_j]}{\langle k_i\rangle},\quad\forall\, i\qquad(24)$$

and

$$\frac{\partial G_i}{\partial\theta_j}=-\frac{\frac{e^{-\theta_j}}{\left(1+e^{-\theta_i-\theta_j}\right)^2}}{\sum_{\substack{j=1\\(j\neq i)}}^N\left(\frac{e^{-\theta_j}}{1+e^{-\theta_i-\theta_j}}\right)}=-\frac{\frac{e^{-\theta_i-\theta_j}}{\left(1+e^{-\theta_i-\theta_j}\right)^2}}{\sum_{\substack{j=1\\(j\neq i)}}^N\left(\frac{e^{-\theta_i-\theta_j}}{1+e^{-\theta_i-\theta_j}}\right)}$$
$$=-\frac{{\rm Cov}[k_i,k_j]}{\langle k_i\rangle},\quad\forall\, i,j.\qquad(25)$$

Let us notice that 1) each element of the Jacobian matrix is a continuous function $\mathbb{R}^N \to \mathbb{R}$ and that 2) the following relationships hold

$$\left|\frac{\partial G_i}{\partial \theta_j}\right| \leq 1, \quad \forall\, i, j; \tag{26}$$

unfortunately, however, when multivariate functions are considered, the set of conditions above is not enough to ensure convergence to the fixed point for *any* choice of the initial value of the parameters. What is needed to be checked is the condition $||J_{\mathbf{G}}(\vec{\theta})|| < 1$, with J indicating the Jacobian of the map (i.e. the matrix of the first, partial derivatives above) and $||.||$ any natural matrix norm: the validity of such a condition has been numerically verified case by case.
